# Angular Insertion Depth for Cochlear Implantation: A Comparative Analysis on Precision of CT, MRI, and x‐Ray

**DOI:** 10.1002/ohn.70248

**Published:** 2026-04-28

**Authors:** Tobias Rader, Hanna Bühler, Matthias P. Fabritius, Martin Canis, Jennifer L. Spiegel

**Affiliations:** ^1^ Department of Otorhinolaryngology LMU University Hospital Ludwig Maximilian University of Munich Munich Germany; ^2^ Department of Radiology LMU University Hospital Ludwig Maximilian University of Munich Munich Germany; ^3^ Department of Otolaryngology–Head & Neck Surgery University of Toronto Toronto Ontario Canada; ^4^ Department of Otolaryngology–Head & Neck Surgery University of Tübingen Medical Center Tübingen Germany

**Keywords:** cochlear coverage, cochlear implant, OTOPLAN, radioanatomy, Stenvers x‐ray

## Abstract

**Objective:**

Personalized medicine in cochlear implantation has advanced significantly with the advent of software tools that allow for detailed analysis of cochlear anatomy. This facilitates the selection of electrode arrays tailored to the individual cochlear duct length (CDL) and supports anatomy‐based fitting strategies. Concurrently, various imaging modalities—both preoperatively and postoperatively—can be assessed in a plug‐and‐play manner. However, to date, no study has performed a direct, systematic comparison of all commonly used imaging modalities preoperative and postoperative computer tomography (CT), magnetic resonance imaging (MRI), and x‐ray.

**Study Design:**

Retrospective study.

**Setting:**

Tertiary Referral Center.

**Methods:**

In total, 31 cochlear implantations with preoperative high‐resolution CT and MRI, and postoperative high‐resolution CT were analyzed with the software OTOPLAN, as well as manual measures from Stenvers view x‐ray. Cochlear anatomy values derived from the different modalities were compared and correlated.

**Results:**

Postoperative CT and x‐ray showed excellent correlation for angular insertion depth across all electrode contacts. In contrast, apical contact values for both preoperative CT and MRI deviated significantly from those derived from postoperative CT scans.

**Conclusion:**

This study is the first to directly correlate intracochlear electrode position estimates obtained from these four imaging modalities and observed a high correlation between postoperative CT and x‐ray measurements across all electrode contacts. In contrast, notable variations were observed when comparing these postoperative results with preoperative imaging data. These findings highlight the potential of postoperative x‐ray‐based analysis as a feasible and efficient method for anatomy‐based fitting, supporting its integration into routine clinical workflows.

Since hearing loss is a major global health concern affecting 1.5 billion people and rising numbers are to be expected due to an aging population,^1^ significance for cochlear implantation (CI) is rising as well. With expanding criteria for CI pertaining to recipients with residual hearing, and an older growing population, novelties in the research field of CI develop in several different directions. Over the years, many advancements have been made on the engineering side, improving coding strategies and the technical finesse of the implant. Surgically, soft surgery techniques are performed to optimally preserve intracochlear structures, as well as the application of different drugs topically or intracochlearly is attempted.^2^, ^3^, ^4^, ^5^, ^6^, ^7^, ^8^, ^9^, ^10^ However, patients' cochlear anatomy, especially in terms of length of the actual cochlear duct, is variable,^11^ and hence, a variety of electrode arrays with different lengths are available to accommodate and tailor to the patients' needs and anatomy.^12^, ^13^, ^14^, ^15^ Measuring the cochlear duct length (CDL) and estimating the angular insertion depth (AID) for different electrode arrays have evolved into a widely adopted approach, facilitated by software that automatically identifies anatomical landmarks on temporal bone computed tomography (CT) scans and calculates the relevant parameters.^16^, ^17^, ^18^, ^19^, ^20^, ^21^, ^22^ Novel concepts for activation and fitting of the CI now involve anatomy‐based fitting (ABF) by applying the Greenwood function.^23^, ^24^ To do so, a postoperative CT scan warrants the measurement of the real AID and the frequency range each electrode contact is located.^25^ Different groups have attempted to change the CT protocol to minimize radiation exposure to the patient,^26^, ^27^ which is essential especially in the very young CI recipients. Radiation of the brain can cause malignant conversion and generate brain tumors.^28^, ^29^ MRI has been shown to be viable for CDL estimation preoperatively^30^ but cannot be performed *post‐implantation* due to the artifact shadow of the CI magnet in the receiver stimulator unit. Over decades, postoperative assessment of the electrode array location has been done with the Stenvers view x‐ray, which has shown quite an accurate estimation of the AID.^31^, ^32^ This study evaluates the measured AIDs on different imaging modalities and correlates them with measurements on postoperative CT scans and x‐ray in the Stenvers view.

## Material and Methods

### Study Design, Ethical Considerations, and Patient Selection

This retrospective study was conducted at a tertiary referral center in accordance with the ethical principles of the 2002 Declaration of Helsinki and approved by the local Review Board and Ethics Committee (Ethikkommission der Medizinischen Fakultät der Ludwig‐Maximilians‐Universität München; reference number 19‐562). Patients who underwent CI between September 2019 and April 2023 and had available preoperative and postoperative CT scans, a preoperative MRI scan, and a postoperative Stenvers view x‐ray were screened for inclusion. Inclusion criteria comprised the following: (1) CT slice thickness of the temporal bone <0.7 mm, (2) absence of inner or middle ear malformations, and (3) complete electrode array insertion (see Supplemental Figure S1, available online).

### Measurements of AID in CT and MRI Data Sets

Preoperative CT and MRI data sets, as well as postoperative CT scans, were uploaded into *OTOPLAN* version 4.0 (CE‐certification number: G1 17 10 95657 003), developed by CAScination AG (available at: https://www.cascination.com/products/otoplan. Accessed April 8, 2024).

**Figure 1:**
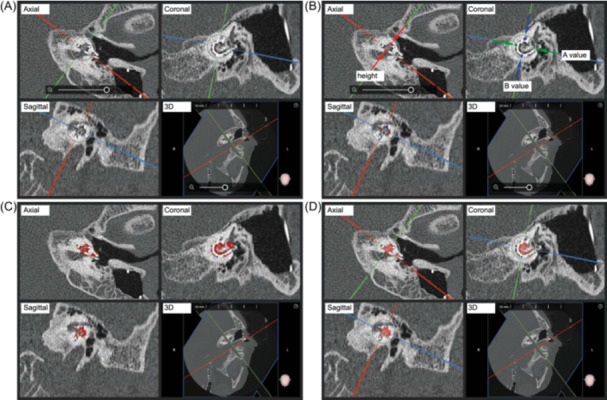
Measurement of cochlear duct length. (A) Axial computer tomography (CT) image of the temporal bone. (B) In the coronal view, the *A*‐value (green arrows) and the *B*‐value (blue arrows) are shown. (C) The red overlay indicates the automated detection of the electrode array. (D) Visualization of the software's automated identification.

Three‐dimensional reconstructions from CT and MRI data were analyzed using the automated functions of *OTOPLAN* version 4.0. The software enables precise identification of relevant cochlear structures and predictive modeling of electrode array positioning. CDL was measured using multiplanar reconstruction. Measurements were conducted as previously described.^11^ Briefly, the plane displaying the complete basal turn of the cochlea was reconstructed to determine the *A*‐value, defined as the largest distance from the round window (RW) to the contralateral cochlear wall; the *B*‐value, defined as the perpendicular distance between the cochlear walls; and the *H*‐value, representing cochlear height, measured in an orthogonal plane (Figure 1). The software then computes the CDL using the elliptic‐circular approximation (ECA) method^33^ and derives the AID and cochlear place frequency according to the Greenwood function,^34^ relative to the selected electrode. Based on these parameters, a three‐dimensional cochlear model is automatically generated. For predictive estimation of the electrode array position, the corresponding electrode type is selected by the investigator according to the measured CDL and projected into the 3D reconstruction.

For postoperative CT scans of the temporal bone, a slice thickness below 0.7 mm was used. Measurements were performed analogously to those described above. After selecting the implant type and electrode array, individual electrode contacts were automatically detected within the cochlea, and the AID for each contact was calculated. All automated detections could be reviewed and manually adjusted by the user as required.

### Measurements of Insertion Depth in Postoperative x‐Ray

**Figure 2:**
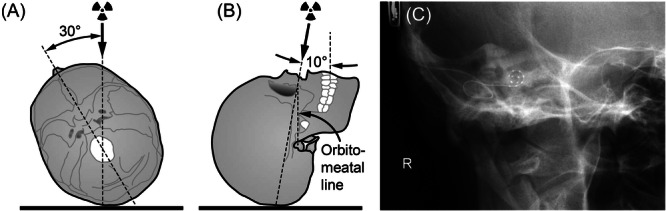
Modified Stenvers projection. (A) Dotted lines illustrate the 30° rotation of the x‐ray beam in the axial and (B) the 10° cranio‐caudal angulation in the sagittal plane. (C) Representative radiograph obtained using the modified Stenvers view.

When analyzing the Stenvers view x‐ray images, the electrode array was first evaluated for correct placement, specifically with respect to potential tip fold‐over or kinking, before proceeding to the measurement of the AID. A modified Stenvers projection was used for radiographic imaging. The examination is performed with the patient in a supine position, using an anteroposterior (A.P.) beam direction. The head is positioned according to the auriculo‐orbital plane and rotated approximately 30° toward the contralateral side. The x‐ray tube is then angled 10° in a cranio‐caudal direction. The central beam is centered on the ipsilateral canthus and collimated to include the nasal root and the external auditory canal. This modified orientation aligns the beam with the cochlear modiolar axis, thereby optimizing the visualization of the osseous labyrinthine structures (Figure 2). All images were obtained using a Siemens Ysio Max digital radiography system (Siemens Healthineers) equipped with an anti‐scatter grid. For pediatric patients, imaging parameters were set to 70 kV with automatic exposure control (AEC) activated in the central measuring chamber and a 0.2‐mm copper filter applied. For adult patients, exposure settings were 77 kV with AEC and no additional filtration. In all cases, exposure parameters were automatically optimized to ensure diagnostic image quality while maintaining adherence to radiation dose minimization principles.

**Figure 3:**
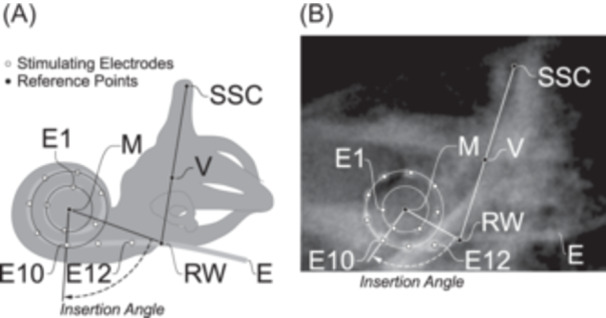
Measurements of angular insertion depth on x‐ray. (A) Schematic measurement; (B) Stenvers view x‐ray. Angle is measured (dotted arrow) starting from the line modiolus (M) to the round window (RW). E1‐E12, electrode contacts; SSC, superior semicircular canal; V, vestibule.

The paradigm to measure the AID was adapted from Xu et al^35^ and Rader et al.^32^ As illustrated in Figure 3, a line connecting the superior semicircular canal (SCC) and vestibule (V) was extended toward the electrode array at the level of the RW. Each electrode contact was identified and numbered from apical (E1) to basal (E12). The modiolus was estimated at the midpoint between the basal and middle turns of the cochlea, and a reference line was drawn connecting the RW and the modiolus. Angular measurements were performed using Angle Meter 360 (version 1.9; Alexey Koslov). The reference line between the modiolus and the RW was defined as 0°.^36^ The AID for each electrode contact in the basal turn was measured directly, while for electrodes located in the second turn, 360° was added to the measured angle (Figure 3).

For this comparative study, all included patients were implanted with electrode arrays from the same manufacturer (MED‐EL GmbH), each comprising 12 electrode contacts.

### Statistical Analysis

All statistical analyses, as well as the generation of tables and figures, were performed using *RStudio* (Posit Software, PBC; Version 4.3.2) (Posit Team, 2025) and *Microsoft Excel* (Version 16.99.2; Microsoft Corporation). Data normality was assessed using *Q*‐*Q* plots. As the majority of data sets demonstrated normal distribution, a one‐way analysis of variance (ANOVA) was applied. Post hoc comparisons were conducted using Tukey's honestly significant difference (HSD) test.

## Results

### Demographic Data and Cochlear Measurements

Of the 108 ears with available postoperative CT scans, 31 met the inclusion criteria for this study (Supplemental Figure S1, available online). Sixteen participants (51.6%) were female, and 16 (51.6%) were implanted on the right ear. The mean age at implantation was 45.48 ± 26.91 years (range: 0.67‐86.19 years).

#### Comparison of Cochlear Measurements Between Postoperative CT and Preoperative Modalities

The estimated CDL was significantly underestimated in preoperative imaging compared to postoperative CT measurements. Specifically, the CDL obtained from preoperative CT (34.46 ± 1.86 mm; *P* < .001) and preoperative MRI (35.87 ± 2.17 mm; *P* < .001) were lower than those from postoperative CT (38.06 ± 2.26 mm). This pattern was consistent for the CDL of the full organ of corti (preoperative CT: 34.46 ± 1.87 mm; *P* < .001; preoperative MRI: 35.87 ± 2.16 mm; *P* < .001; postoperative CT: 38.06 ± 2.25 mm) and for measurements excluding the hook region (preoperative CT: 31.96 ± 1.87 mm; *P* < .001; preoperative MRI: 33.37 ± 2.16 mm; *P* < .001; postoperative CT: 35.57 ± 2.24 mm).

*A*‐values were similar between preoperative CT (9.05 ± 0.44 mm) and preoperative MRI (9.08 ± 0.46 mm; *P* = .960). *B*‐values were slightly higher in preoperative MRI (6.84 ± 0.50 mm) than in preoperative CT (6.47 ± 0.40 mm; *P* = .005), while cochlear height did not differ significantly between modalities (preoperative CT: 4.00 ± 0.30 mm; preoperative MRI: 3.99 ± 0.30 mm; *P* = .070). Overall, cochlear measurements were significantly greater in postoperative CTs (*A*‐value: 9.41 ± 0.55 mm; *P* = .012; *B*‐value: 7.30 ± 0.50 mm; *P* < .001; height: 4.18 ± 0.40 mm; *P* = .012) compared to preoperative imaging. A detailed summary of all measured parameters across modalities is provided in Table 1.

**Table 1:** Cochlear Parameters Across All Modalities: Preoperative Computer Tomography (CT), Preoperative Magnetic Resonance Imaging (MRI), Postoperative CT, and Postoperative Stenvers x‐Ray

	CT pre	MRI pre	CT post	Stenvers
	mean ± SD	mean ± SD	mean ± SD	mean ± SD
*A*‐value (mm)	9.05 ± 0.44	9.08 ± 0.46	9.41 ± 0.545	n/a
*B*‐value (mm)	6.47 ± 0.36	6.84 ± 0.45	7.30 ± 0.493	n/a
height (mm)	4.00 ± 0.34	3.99 ± 0.35	4.19 ± 0.411	n/a
Estimated CDL (mm)	34.46 ± 1.86	35.87 ± 2.17	38.06 ± 2.258	n/a
CDL full OC (mm)	34.46 ± 1.87	35.87 ± 2.16	38.06 ± 2.248	n/a
CDL‐OC from 0 (mm)	31.96 ± 1.87	33.37 ± 2.16	35.57 ± 2.245	n/a
AID_E1 (degree)	670.76 ± 78.38	634.30 ± 81.20	678.67 ± 96.42	696.02 ± 94.84
AID_E2 (degree)	593.58 ± 68.43	561.81 ± 71.85	597.02 ± 87.33	613.42 ± 89.52
AID_E3 (degree)	520.63 ± 61.48	491.82 ± 64.70	522.05 ± 89.53	526.29 ± 84.89
AID_E4 (degree)	448.24 ± 52.44	423.45 ± 55.14	442.93 ± 80.88	450.10 ± 74.68
AID_E5 (degree)	379.46 ± 41.41	359.84 ± 43.71	365.94 ± 58.86	372.15 ± 53.97
AID_E6 (degree)	318.42 ± 31.53	303.51 ± 33.69	309.44 ± 48.16	316.63 ± 46.22
AID_E7 (degree)	264.86 ± 24.76	253.15 ± 26.85	261.26 ± 43.21	263.26 ± 42.20
AID_E8 (degree)	214.93 ± 19.87	205.51 ± 21.72	215.09 ± 40.59	214.21 ± 37.97
AID_E9 (degree)	167.01 ± 14.66	160.12 ± 16.13	167.19 ± 39.94	169.22 ± 33.22
AID_E10 (degree)	123.25 ± 10.06	118.66 ± 11.02	120.46 ± 38.31	122.72 ± 32.50
AID_E11 (degree)	84.05 ± 6.55	81.40 ± 6.75	76.77 ± 34.68	72.80 ± 32.86
AID_E12 (degree)	48.97 ± 4.57	47.90 ± 3.43	41.00 ± 25.40	30.43 ± 24.01

Abbreviations: AID, angular insertion depth; CDL, cochlear duct length; E1‐12, electrode contacts 1 to 12; OC, organ of corti; SD, standard deviation.

### Correlation of AID Between the Different Modalities

Assuming the postoperative CT scan represents the reference standard (“true measure”) for electrode insertion depth, corresponding values from all other modalities were compared to it.

#### Correlation Between Postoperative CT Scan and Postoperative x‐Ray

**Figure 4:**
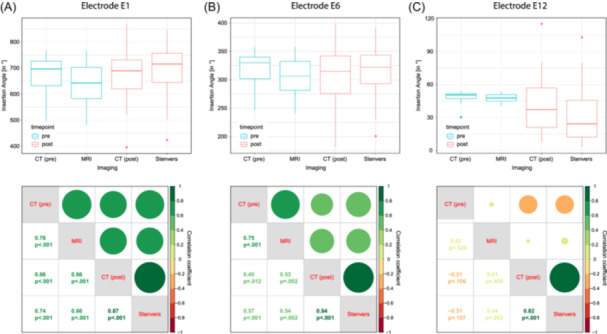
Correlation of angular insertion depth. The upper row shows boxplots for (A) representative apical electrode contact E1, (B) representative middle electrode contact E6, and (C) representative basal electrode contact E12. Preoperative imaging modalities are indicated in green (computer tomography [CT] and magnetic resonance imaging [MRI]), and postoperative modalities in red (CT and Stenvers view x‐ray). The lower row illustrates the correlation between each modality for the corresponding electrode contacts (A) E1, (B) E6, and (C) E12. Boxplots depict the median as a horizontal line and outliers as small circles.

A strong and statistically significant correlation was observed between postoperative CT and Stenvers view x‐ray measurements across all electrode contacts: apical (E1: *r* = 0.87; *P* < .001), middle (E6: *r* = 0.84; *P* < .001), and basal (E12: *r* = 0.82; *P* < .001; Figure 4).

#### Correlation Between Postoperative CT Scan and Preoperative CT and MRI Scans

Lower correlations were identified for preoperative CT and MRI compared to postoperative CT (see Figure 4 for *r*‐ and *P*‐values). Nevertheless, less variability was observed for basal electrode contacts in preoperative imaging, likely due to the closer anatomical proximity to the RW.

#### Deviation of AID Compared Between Postoperative CT and All Other Modalities

**Figure 5:**
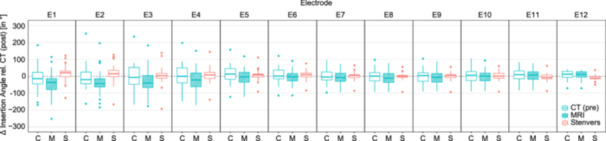
Delta values calculated between the “true” angular insertion depth derived from postoperative computer tomography (CT) and each other modality: preoperative CT (C, green), preoperative magnetic resonance imaging (MRI) (M, green), and postoperative Stenvers view x‐ray (S, red). Boxplots are shown for each electrode contact (E1‐E12). Boxplots depict the median as a horizontal line and outliers as small circles. The dotted line indicates zero difference.

Differences in AID between postoperative CT and all other modalities were calculated for each electrode contact (E1‐E12). Greater deviations were observed for apical contacts and in preoperative imaging modalities, although these differences did not reach statistical significance (*P* = .450; Figure 5).

## Discussion

This is the first study directly correlating intracochlear electrode estimations obtained from four different imaging modalities: (1) preoperative MRI, (2) preoperative CT, and (3) postoperative Stenvers view x‐ray measurements with (4) postoperative CT. An overview of the current literature on comparing cochlear analysis on different imaging modalities is given in Supplemental Table S1, available online. In our study, values deriving from postoperative CT scan and x‐ray correlated extremely well across all electrode contacts. Both preoperative CT and MRI had good correlation for basal contacts, but significantly deviated from apical contacts, which we associate with the farther proximity to the RW.

The limitations of this study are primarily related to its retrospective design and single‐center setting, as well as the relatively small sample size of 31 ears. At our institution, postoperative CT scans are not routinely performed to assess electrode array positioning, given the higher radiation exposure compared to Stenvers view radiographs. Consequently, CT data were only available for cases in which electrode dislocation was clinically suspected or when secondary imaging was indicated for other reasons (see Supplemental Figure S1, available online). To ensure a homogeneous cohort and to validate the measurement method in anatomically normal conditions, all patients with inner ear malformations were excluded. Considering the limited sample size (n = 31) of this single‐center study, larger multicenter cohorts are warranted to enhance the reproducibility and generalizability of the manual x‐ray AID measurement approach. Furthermore, the development of an automated measurement algorithm could minimize interobserver variability and reduce potential measurement errors. Additional limitations pertain to imaging quality, particularly regarding the resolution and technical constraints of CT and MRI scans.

Software‐based analysis and measurement of the cochlea preoperatively is a widely adopted concept to select the individually tailored electrode array to the patient and has been well‐studied over the last few years^37^ (Supplemental Table S2, available online). With the technical advancement of the software over the years, automated measurements on high‐resolution CT scans were enabled, as well as performing measurements on MRI scans. The measurements/estimations from the preoperative CT scans in our study (34.46 ± 1.86 mm, range: 30.60‐38.20 mm) show comparable mean‐values and range to previous studies.^37^ However, the use of different resolutions and slice thickness, as well as different versions of the software, may result in variability of the calculated values.^37^ Good correlation of preoperative CT and MRI‐scan estimates was found in our study (35.87 ± 2.17 mm; range 32.30‐41.90 mm), which has been confirmed by other groups as well.^30^, ^38^, ^39^ The group around Weber found preoperative MRI images to reveal slightly longer CDL values in comparison to preoperative CT scans^39^ (*mean difference* = 0.89 mm; 95% confidence interval 0.50‐1.28). Since software development has advanced to automated analysis of postoperative CT scans, the exact location of each electrode contact can be identified, which has allowed the evolvement of ABF. The individually tailored frequency allocation to the cochlea allows to mitigate electrode‐to‐frequency mismatch and with improved speech discrimination.^40^, ^41^ Our study results revealed a deviation of preoperative to postoperative obtained CT measurements similar to the results of Mueller‐Graff et al, who recommended high‐resolution preoperative scans to mitigate this effect.^25^, ^42^, ^43^ This deviation and underestimation in the preoperatively obtained values may be attributed to algorithmic bias in the automated calculation process, as the software relies on the RW as a fixed anatomical reference point for deriving measurements.

Recent work has studied the eligibility of postoperative x‐ray for ABF. The group around Gallant has demonstrated a strong linear correlation between the AID obtained from CT scan and x‐ray in a cadaveric study.^44^ This was confirmed by Alahmadi et al who found values calculated from the x‐ray well‐correlating with those acquired from postoperative CT scans, with both manually measuring the AID on the x‐ray with the DICOM viewer,^45^ as well as automated measurements with OTOPLAN version 5.^46^ Interestingly, a deviation of correlation was found for the E6 contact, possibly explained by the transition from first to second turn. Depending on the angle at which the x‐ray was shot and the alignment of the temporal bone structures on the x‐ray plan differentiation of the respective electrode contacts could be compromised. Our study did not find a deviation of the E6 electrode but in contrast found excellent correlation across all contacts (Figure 4), but with lower values (0.82) for the most apical E12. These results underscore the value and versatility of a postoperative Stenvers x‐ray, where the electrode contacts were well illustrated, and therefore AID can be easily derived from. These values can then be further utilized for an individually tailored ABF concepts involving ABF which opens the door to pivot from CT to x‐ray for ABF. Moreover, x‐ray imaging is a quick and easily accessible procedure that can be performed immediately postoperatively in the operating room. It streamlines clinical workflow, is more cost‐effective than CT imaging, and exposes the patient to a substantially lower dose of ionizing radiation. In addition, the accuracy of assessing postoperative CT scans can be reduced by metal artifacts.^47^ However, x‐rays are not without significant limitation in a clinical setting, subject to a lower spatial resolution compared to CT scans, which might lead to oversight of electrode‐related problems. The quality and interpretation depend on the radiology technician's and reader's experience and might also differ between the different planes or views that are selected for the investigation.^48^, ^49^ Going forward, these confounders can be mitigated by including larger cohorts and gaining more experience performing ABF from x‐ray‐derived values.

## Conclusion

This is the first study directly correlating intracochlear electrode estimations of four different imaging modalities: (1) preoperative MRI, (2) preoperative CT, and (3) postoperative Stenvers x‐ray measurements with (4) postoperative CT. It shows good correlation of postoperative CT scan and x‐ray AID values across all electrode contacts, which underscores the feasibility of ABF from postoperative x‐ray‐derived analysis. In contrast, apical contact values for both preoperative CT and MRI deviated significantly from those derived from postoperative CT scan, which we associate with the farther proximity to the RW.

## Authors' Note

Consent to participate: Retrospective study, no consent required.

Consent for publication (include appropriate statements): Retrospective study, no consent required.

Consolidated standard of reporting trial statement: Retrospective study, not required.

## Author Contributions

**Tobias Rader**, conceptualization, methodology, project administration, data curation, investigation, visualization, supervision, writing—original draft.; **Hanna Bühler**, date collection, data curation, formal analysis, visualization, writing—original draft; **Matthias P. Fabritius**, methodology, writing—original draft; **Martin Canis**, validation, writing—review and editing; **Jennifer L. Spiegel**, conceptualization, project administration, data collection, data curation, investigation, supervision, writing— original draft.

## Disclosures

### Competing interests

Both Tobias Rader and Jennifer L. Spiegel received travel expenses from MED EL, Innsbruck, Austria.

### Funding source

This study was not supported by funding.

## Supporting information


**Supplemental Figure S1: Consolidated Standard Reporting of Trials (CONSORT flow diagram).** Screened and included patients with reasons for exclusion.

**Supplemental Table S1: Overview of the current literature on comparing cochlear analysis on different imaging modalities.** CT, computer tomography; fpVCT; flat‐panel volume CT; MRI, magnetic resonance imaging; msCT, multislice computer tomography; SECO, secondary reconstruction.

**Supplemental Table S2: Overview of literature on software‐based cochlear analysis.** CBCT, cone‐beam computer tomography; CT, computer tomography; fpVCT; flat‐panel volume CT; MRI, magnetic resonance imaging; msCT, multislice computer tomography; N/G, not given; SECO, secondary reconstruction.

## Data Availability

Original data is available upon reasonable request. No custom code was used to process the data described in the manuscript.
