# Supplementary material for: Angular Insertion Depth for Cochlear Implantation: A Comparative Analysis on Precision of CT, MRI, and x‐Ray
Source: Otolaryngol Head Neck Surg. 2026 Apr 28;175(1):214–22. doi: 10.1002/ohn.70248 (PMC13327508; doi:10.1002/ohn.70248)
Supplement: Supplementary file 1 — Supplemental Figure S1: Consolidated Standard Reporting of Trials (CONSORT flow diagram). Screened and included patients with reasons for exclusion. [file OHN-175-214-s001.pdf]

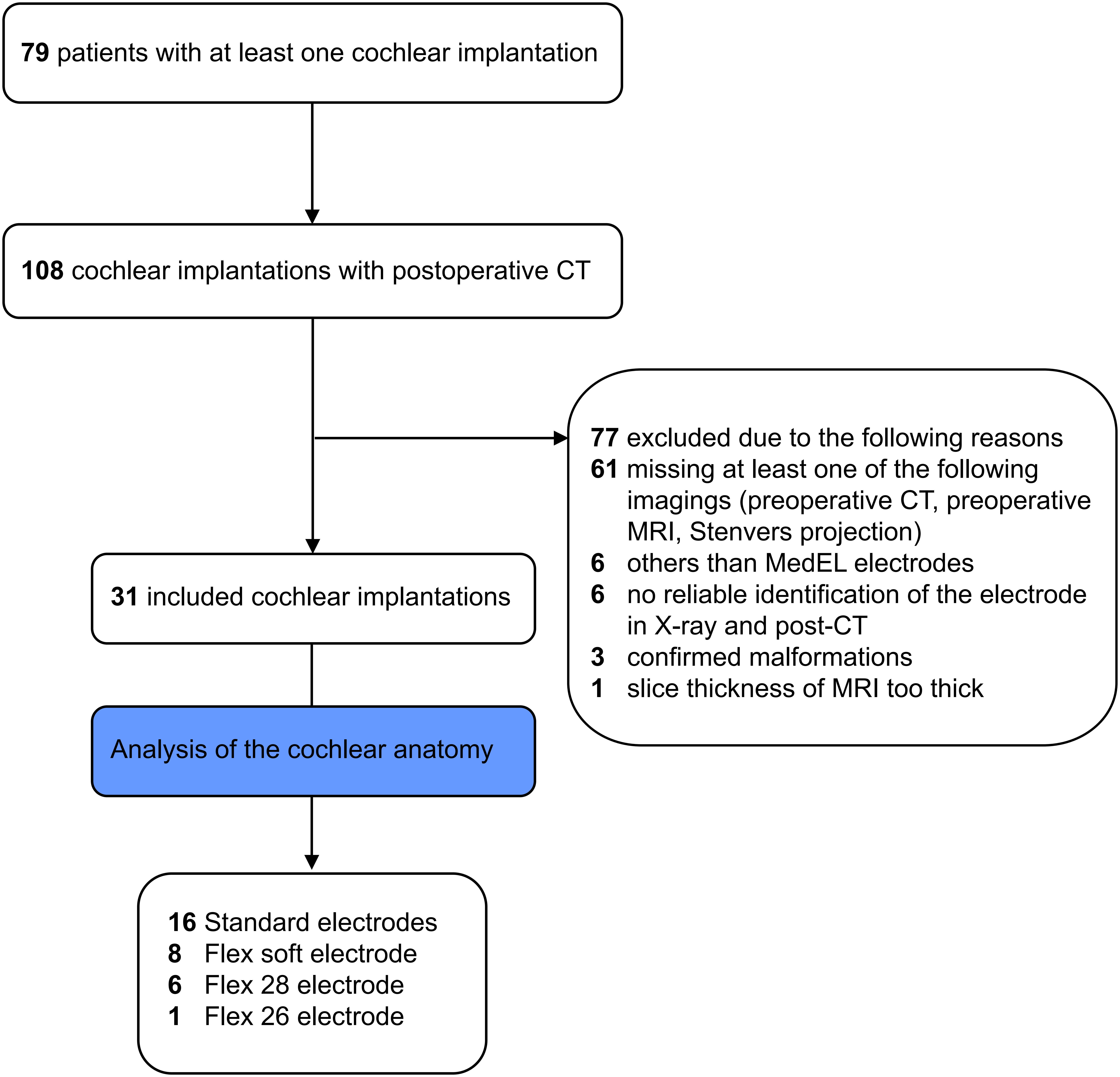

79 patients with at least one cochlear implantation
108 cochlear implantations with postoperative CT
77 excluded due to the following reasons
61 missing at least one of the following imagings (preoperative CT, preoperative MRI, Stenvers projection)
6 others than MedEL electrodes
6 no reliable identification of the electrode in X-ray and post-CT
3 confirmed malformations
1 slice thickness of MRI too thick
31 included cochlear implantations
Analysis of the cochlear anatomy
16 Standard electrodes
8 Flex soft electrode
6 Flex 28 electrode
1 Flex 26 electrode
