# Supplementary material for: Angular Insertion Depth for Cochlear Implantation: A Comparative Analysis on Precision of CT, MRI, and x‐Ray
Source: Otolaryngol Head Neck Surg. 2026 Apr 28;175(1):214–22. doi: 10.1002/ohn.70248 (PMC13327508; doi:10.1002/ohn.70248)
Supplement: Supplementary file 2 — Supplemental Table S1: Overview of the current literature on comparing cochlear analysis on different imaging modalities. CT, computer tomography; fpVCT; flat‐panel volume CT; MRI, magnetic resonance imaging; msCT, multislice computer tomography; SECO, secondary reconstruction. [file OHN-175-214-s003.pdf]

Supplemental Table 1:

Year	Author	Modality	CT preop	CT postop	MRI preop	X-ray postop	Measurement	N
2020	George-Jones et al. [37]	CT<br>MRI	yes	no	yes	no	OTOPLAN V2	27
2021	Mlynski et al. [21]	CT<br>x-ray	yes	yes	no	yes	OTOPLAN V2	59
2022	Weber et al. [39]	CT<br>MRI	yes	no	yes	no	OTOPLAN V3	20
2022	Ricci et al. [50]	CT<br>MRI<br>x-ray	yes	yes	yes	yes	OTOPLAN	1
2023	Mueller-Graff et al. [25]	CT<br>msCT<br>fpVCT<br>fpVCT+SECO	yes	yes	no	no	OTOPLAN V2	10
2023	Otte et al. [30]	MRI<br>CT	yes	no	yes	no	OTOPLAN V2	44
2023	Thomas et al. [51]	CT<br>MRI	yes	no	yes	no	OTOPLAN V2	78
2024	Alahmadi et al. [45]	CT<br>x-ray	yes	yes	no	yes	OTOPLAN V3	53
2024	Alahmadi et al. [46]	CT<br>x-ray	no	yes	no	yes	OTOPLAN V5	50
2025	Rader et al. [(this article)]	CT<br>MRI<br>x-ray	yes	yes	yes	yes	OTOPLAN V4	31

References:

21. Mlynski R, Lusebrink A, Oberhoffner T, Langner S, Weiss NM. Mapping Cochlear Duct Length to Electrically Evoked Compound Action Potentials in Cochlear Implantation. *Otol Neurotol*. Mar 1 2021;42(3):e254-e260. doi:10.1097/MAO.0000000000002957

25. Muller-Graff FT, Voelker J, Kurz A, Hagen R, Neun T, Rak K. Accuracy of radiological prediction of electrode position with otological planning software and implications of high-resolution imaging. *Cochlear Implants Int*. May 2023;24(3):144-154. doi:10.1080/14670100.2022.2159128

30. Otte MS, Mueller V, Burkhardt P, et al. Cochlear measurement in computed tomography and magnetic resonance imaging data sets by the Otoplan measurement tool: a retrospective comparative study. *J Laryngol Otol*. Aug 2024;138(8):869-873. doi:10.1017/S0022215124000239

37. Muller-Graff FT, Spahn B, Herrmann DP, et al. Comprehensive literature review on the application of the otological surgical planning software OTOPLAN(R) for cochlear implantation. *HNO*. Jun 11 2024;Umfassender Literaturuberblick uber die Anwendung der otologisch-chirurgischen Planungssoftware OTOPLAN(R) bei der Cochleaimplantation. Englische Version. doi:10.1007/s00106-023-01417-4

39. Weber L, Kwok P, Picou EM, Wendl C, Bohr C, Marcrum SC. [Measuring the cochlea using a tablet-based software package: influence of imaging modality and rater background]. *HNO*. Oct 2022;70(10):769-777. Vermessung der Cochlea mittels eines Tablet-basierten Softwarepakets: Einfluss der Bildgebungsmodalitat und des Untersucherhintergrunds. doi:10.1007/s00106-022-01208-3

45. Alahmadi A, Abdelsamad Y, Thabet EM, et al. Advancing Cochlear Implant Programming: X-ray Guided Anatomy-Based Fitting. *Otol Neurotol*. Feb 1 2024;45(2):107-113. doi:10.1097/MAO.0000000000004069

46. Alahmadi A, Abdelsamad Y, Hafez A, Hagr A. X-ray guided anatomy-based fitting: The validity of OTOPLAN. *PLoS One*. 2024;19(11):e0313567. doi:10.1371/journal.pone.0313567

50. Ricci G, Lapenna R, Gambacorta V, Della Volpe A, Faralli M, Di Stadio A. OTOPLAN, Cochlear Implant, and Far-Advanced Otosclerosis: Could the Use of Software Improve the Surgical Final Indication? *J Int Adv Otol*. Jan 2022;18(1):74-78. doi:10.5152/iao.2022.21329

51. Thomas JP, Klein H, Haubitz I, Dazert S, Volter C. Intra- and Interrater Reliability of CT- versus MRI-Based Cochlear Duct Length Measurement in Pediatric Cochlear Implant Candidates and Its Impact on Personalized Electrode Array Selection. *J Pers Med*. Apr 4 2023;13(4). doi:10.3390/jpm13040633
