# Supplementary material for: Angular Insertion Depth for Cochlear Implantation: A Comparative Analysis on Precision of CT, MRI, and x‐Ray
Source: Otolaryngol Head Neck Surg. 2026 Apr 28;175(1):214–22. doi: 10.1002/ohn.70248 (PMC13327508; doi:10.1002/ohn.70248)
Supplement: Supplementary file 3 — Supplemental Table S2: Overview of literature on software‐based cochlear analysis. CBCT, cone‐beam computer tomography; CT, computer tomography; fpVCT; flat‐panel volume CT; MRI, magnetic resonance imaging; msCT, multislice computer tomography; N/G, not given; SECO, secondary reconstruction. [file OHN-175-214-s002.pdf]

Supplemental Table 2:

Year	Author	Modality	CT preop	CT postop	MRI preop	X-ray postop	Other	Measurement	N
2018	Lovato et al. [52]	CT	yes	no	no	no	no	OTOPLAN	1
2020	Almuhawas et al. [53]	CT	yes	no	no	no	no	OTOPLAN	92
2020	Andersen et al. [54]	CT	yes	no	no	no	no	ITK-SNAP<br>OTOPLAN<br>Fiji<br>Elastix	18
2020	George-Jones et al. [37]	CT<br>MRI	yes	no	yes	no	no	OTOPLAN	27
2020	Khurayzi et al. [55]	CT	yes	no	no	no	no	OTOPLAN	88
2020	Lovato et al. [56]	CT	yes	no	no	no	no	OTOPLAN	5
2020	Topsakal et al. [57]	CT	yes	no	no	no	no	OTOPLAN	N/G
2021	Aljazeeri et al. [58]	CT	yes	no	no	no	no	OTOPLAN	169
2021	Auinger et al. [59]	CT	yes	no	no	no	no	OTOPLAN	100
2021	Chen et al. [60]	CT	yes	no	no	no	no	OTOPLAN	68
2021	Cooperman et al. [61]	CT	yes	no	no	no	no	OTOPLAN	166
2021	Jablonski et al. [62]	CBCT	yes	yes	no	no	Temporal bones	OTOPLAN	16
2021	Lee et al. [63]	CT	yes	yes	no	no	no	OTOPLAN	51
2021	Lo Russo et al. [64]	fpVCT	no	yes	no	no	no	Horos	50
2021	Mlynski et al. [21]	CT<br>x-ray	yes	yes	no	yes	no	OTOPLAN	59
2021	Mueller et al. [65]	CBCT	yes	no	no	no	no	OTOPLAN	8
2021	Spiegel et al. [11]	CT	yes	no	no	no	no	OTOPLAN	108
2021	Taeger et al. [66]	CT<br>MRI	yes	no	yes	no	no	Horos	42
2022	Bhavana et al. [67]	CT	no	yes	no	no	no	OTOPLAN	26
2022	Dhanasingh at al. [68]	CT	yes	no	no	no	no	OTOPLAN	112
2022	Di Maro et al. [69]	HRCT	yes	yes	no	no	no	OTOPLAN	N/G
2022	Dutrieux et al. [70]	CBCT<br>CT	no	yes	no	no	no	OTOPLAN	106
2022	Kurz et al. [71]	High-volume<br>Dyna CT	yes	yes	no	no	no	OTOPLAN	5
2022	Li et al. [72]	CT	yes	no	no	no	no	OTOPLAN	247
2022	Mertens et al. [73]	CT	no	yes	no	no	no	OTOPLAN	39
2022	Müller-Graff et al. [74]	fpVCT<br>SECO<br>msCT	yes	yes	no	no	no	OTOPLAN V2	N/G
2022	Ricci et al. [50]	CT<br>MRI<br>x-ray	yes	yes	yes	yes	no	OTOPLAN	1
2022	Thimsen et al. [75]	msCT	yes	yes	no	no	no	OTOPLAN	38
2022	Topsakal et al. [76]	CBCT	yes	yes	no	no	no	OTOPLAN	25
2022	Weber et al. [39]	MRI<br>fpCT	yes	no	yes	no	no	OTOPLAN	20
2023	Al-Dhamari et al. [77]	CBCT<br>CT<br>MRI	yes	yes	yes	no	no	ACA	217
2023	Alahmadi et al. [78]	CT	yes	no	no	no	no	OTOPLAN	42
2023	Alshalan et al. [79]	CT	yes	no	no	no	no	OTOPLAN	95
2023	Breitsprecher et al. [18]	CT<br>CBCT	yes	yes	no	yes	Temporal bones	OTOPLAN	20
2023	Breitsprecher et al. [80]	CT	yes	yes	no	no	no	OTOPLAN	80

2023	Geiger et al. [81]	CBCT<br>micro-CT	yes	yes	no	no	Temporal bones	Advanced Bionics software	6
2023	Hajr et al. [82]	CT	yes	no	no	no	no	OTOPLAN	25
2023	Hussain et al. [83]	CT	yes	no	no	no	no	web-based tool, Nautilus	1099
2023	Matin-Mann et al. [84]	CBCT	yes	no	no	no	no	customized version of 3D Slicer	20
2023	Mewes et al. [85]	CT<br>DVT	no	yes	no	yes	DVT	IGCIP	50
2023	Müller-Graff et al. [25]	CT<br>msCT<br>fpVCT<br>fpVCT+SECO	yes	yes	no	no	no	OTOPLAN	N/G
2023	Otte et al. [30]	MRI<br>CT	yes	no	yes	no	no	OTOPLAN	44
2023	Paouris et al. [86]	CT	yes	no	no	no	no	OTOPLAN	109
2023	Rader et al. [87]	CT<br>x-ray	yes	no	no	no	no	OTOPLAN	39
2023	Sieber et al. [88]	msCT	yes	no	no	no	publicly available CT scans	OsiriX MD	5
2023	Swarup et al. [89]	MRI<br>CT	yes	no	yes	no	no	Syngovia	94
2023	Tavora-Vieira et al. [90]	CT	yes	no	no	no	no	OTOPLAN	N/G
2023	Thomas et al. [51]	CT<br>MRI	yes	no	yes	no	no	OTOPLAN	78
2023	Weiss et al. [17]	CT	yes	no	no	no	no	OTOPLAN	120
2024	Alahmadi et al. [45]	CT<br>x-ray	yes	yes	no	yes	no	OTOPLAN	53
2024	Alahmadi et al. [46]	CT<br>x-ray	no	yes	no	yes	no	OTOPLAN	50
2024	Alshalan et al. [91]	CT	yes	no	no	no	no	OTOPLAN	162
2024	Dessard et al. [92]	CBCT	no	yes	no	no	no	OTOPLAN	13
2024	Dhanasingh at al. [93]	CT	yes	no	no	yes	no	freeware 3D slicer	N/G
2024	Jun et al. [94]	CT	yes	no	no	no	no	V-works version 4.0	50
2024	Spahn et al. [95]	fpVCT	yes	no	no	no	Temporal bones	Horos	10

References:

11. Spiegel JL, Polterauer D, Hempel JM, Canis M, Spiro JE, Muller J. Variation of the cochlear anatomy and cochlea duct length: analysis with a new tablet-based software. *Eur Arch Otorhinolaryngol*. May 29 2021; doi:10.1007/s00405-021-06889-0

17. Weiss NM, Breitsprecher T, Wozniak M, et al. Comparing linear and non-linear models to estimate the appropriate cochlear implant electrode array length-are current methods precise enough? *Eur Arch Otorhinolaryngol*. Jul 19 2023; doi:10.1007/s00405-023-08064-z

18. Breitsprecher T, Dhanasingh A, Schulze M, et al. CT imaging-based approaches to cochlear duct length estimation-a human temporal bone study. *Eur Radiol*. Feb 2022;32(2):1014-1023. doi:10.1007/s00330-021-08189-x

21. Mlynski R, Lusebrink A, Oberhoffner T, Langner S, Weiss NM. Mapping Cochlear Duct Length to Electrically Evoked Compound Action Potentials in Cochlear Implantation. *Otol Neurotol*. Mar 1 2021;42(3):e254-e260. doi:10.1097/MAO.0000000000002957

25. Muller-Graff FT, Voelker J, Kurz A, Hagen R, Neun T, Rak K. Accuracy of radiological prediction of electrode position with otological planning software and implications of high-resolution imaging. *Cochlear Implants Int*. May 2023;24(3):144-154. doi:10.1080/14670100.2022.2159128

30. Otte MS, Mueller V, Burkhardt P, et al. Cochlear measurement in computed tomography and magnetic resonance imaging data sets by the Otoplan measurement tool: a retrospective comparative study. *J Laryngol Otol*. Aug 2024;138(8):869-873. doi:10.1017/S0022215124000239

37. Muller-Graff FT, Spahn B, Herrmann DP, et al. Comprehensive literature review on the application of the otological surgical planning software OTOPLAN(R) for cochlear implantation. *HNO*. Jun 11 2024;Umfassender Literaturuberblick uber die Anwendung der otologisch-chirurgischen Planungssoftware OTOPLAN(R) bei der Cochleaimplantation. Englische Version. doi:10.1007/s00106-023-01417-4

39. Weber L, Kwok P, Picou EM, Wendl C, Bohr C, Marcrum SC. [Measuring the cochlea using a tablet-based software package: influence of imaging modality and rater background]. *HNO*. Oct 2022;70(10):769-777. Vermessung der Cochlea mittels eines Tablet-basierten Softwarepakets: Einfluss der Bildgebungsmodalitat und des Untersucherhintergrunds. doi:10.1007/s00106-022-01208-3

45. Alahmadi A, Abdelsamad Y, Thabet EM, et al. Advancing Cochlear Implant Programming: X-ray Guided Anatomy-Based Fitting. *Otol Neurotol*. Feb 1 2024;45(2):107-113. doi:10.1097/MAO.0000000000004069

46. Alahmadi A, Abdelsamad Y, Hafez A, Hagr A. X-ray guided anatomy-based fitting: The validity of OTOPLAN. *PLoS One*. 2024;19(11):e0313567. doi:10.1371/journal.pone.0313567

50. Ricci G, Lapenna R, Gambacorta V, Della Volpe A, Faralli M, Di Stadio A. OTOPLAN, Cochlear Implant, and Far-Advanced Otosclerosis: Could the Use of Software Improve the Surgical Final Indication? *J Int Adv Otol*. Jan 2022;18(1):74-78. doi:10.5152/iao.2022.21329

51. Thomas JP, Klein H, Haubitz I, Dazert S, Volter C. Intra- and Interrater Reliability of CT- versus MRI-Based Cochlear Duct Length Measurement in Pediatric Cochlear Implant Candidates and Its Impact on Personalized Electrode Array Selection. *J Pers Med*. Apr 4 2023;13(4). doi:10.3390/jpm13040633

52. Lovato A, de Filippis C. Utility of OTOPLAN Reconstructed Images for Surgical Planning of Cochlear Implantation in a Case of Post-meningitis Ossification. *Otol Neurotol*. Jan 2019;40(1):e60-e61. doi:10.1097/MAO.0000000000002079

53. Almuhawas FA, Dhanasingh AE, Mitrovic D, et al. Age as a Factor of Growth in Mastoid Thickness and Skull Width. *Otol Neurotol*. Jun 2020;41(5):709-714. doi:10.1097/MAO.0000000000002585

54. Andersen SAW, Bergman M, Keith JP, et al. Segmentation of Temporal Bone Anatomy for Patient-Specific Virtual Reality Simulation. *Ann Otol Rhinol Laryngol*. Jul 2021;130(7):724-730. doi:10.1177/0003489420970217

55. Khurayzi T, Almuhawas F, Sanosi A. Direct measurement of cochlear parameters for automatic calculation of the cochlear duct length. *Ann Saudi Med*. May-Jun 2020;40(3):212-218. doi:10.5144/0256-4947.2020.218

56. Lovato A, Marioni G, Gamberini L, Bonora C, Genovese E, de Filippis C. OTOPLAN in Cochlear Implantation for Far-Advanced Otosclerosis. *Otol Neurotol*. Jun 18 2020;doi:10.1097/MAO.0000000000002722

57. Topsakal V, Matulic M, Assadi MZ, Mertens G, Rompaey VV, Van de Heyning P. Comparison of the Surgical Techniques and Robotic Techniques for Cochlear Implantation in Terms of the Trajectories Toward the Inner Ear. *J Int Adv Otol*. Apr 2020;16(1):3-7. doi:10.5152/iao.2020.8113

58. Aljazeeri I, Hamed N, Abdelsamad Y, Sharif T, Al-Momani M, Hagr A. Anatomy-Based Frequency Allocation in Cochlear Implantation: The Importance of Cochlear Coverage. *Laryngoscope*. Nov 2022;132(11):2224-2231. doi:10.1002/lary.30004

59. Auinger AB, Dahm V, Liepins R, Riss D, Baumgartner WD, Arnoldner C. Robotic Cochlear Implant Surgery: Imaging-Based Evaluation of Feasibility in Clinical Routine. *Front Surg*. 2021;8:742219. doi:10.3389/fsurg.2021.742219

60. Chen Y, Chen J, Tan H, et al. Cochlear Duct Length Calculation: Comparison Between Using Otoplan and Curved Multiplanar Reconstruction in Nonmalformed Cochlea. *Otol Neurotol*. Aug 1 2021;42(7):e875-e880. doi:10.1097/MAO.0000000000003119

61. Cooperman SP, Aaron KA, Fouad A, Tran E, Blevins NH, Fitzgerald MB. Assessment of Inter- and Intra-Rater Reliability of Tablet-Based Software to Measure Cochlear Duct Length. *Otol Neurotol*. Apr 1 2021;42(4):558-565. doi:10.1097/MAO.0000000000003015

62. Jablonski GE, Falkenberg-Jensen B, Bunne M, et al. Fusion of Technology in Cochlear Implantation Surgery: Investigation of Fluoroscopically Assisted Robotic Electrode Insertion. *Front Surg*. 2021;8:741401. doi:10.3389/fsurg.2021.741401

63. Lee SY, Jung Bae Y, Carandang M, et al. Modiolar Proximity of Slim Modiolar Electrodes and Cochlear Duct Length: Correlation for Potential Basis of Customized Cochlear Implantation With Perimodiolar Electrodes. *Ear Hear*. Aug 7 2020;doi:10.1097/AUD.0000000000000920

64. Lo Russo F, Conte G, Di Berardino F, et al. Impact of Cochlear Implant Array Placement on Speech Perception. *Clin Neuroradiol*. Mar 2022;32(1):175-183. doi:10.1007/s00062-021-01046-w

65. Mueller F, Hermann J, Weber S, O'Toole Bom Braga G, Topsakal V. Image-Based Planning of Minimally Traumatic Inner Ear Access for Robotic Cochlear Implantation. *Front Surg*. 2021;8:761217. doi:10.3389/fsurg.2021.761217

66. Taeger J, Muller-Graff FT, Ilgen L, et al. Cochlear Duct Length Measurements in Computed Tomography and Magnetic Resonance Imaging Using Newly Developed Techniques. *OTO Open*. Jul-Sep 2021;5(3):2473974X211045312. doi:10.1177/2473974X211045312

67. Bhavana K, Timmaraju S, Kumar V, et al. OTOPLAN-Based Study of Intracochlear Electrode Position Through Cochleostomy and Round Window in Transcanal Veria Technique. *Indian J Otolaryngol Head Neck Surg*. Dec 2022;74(4):575-581. doi:10.1007/s12070-022-03228-5

68. Dhanasingh AE, Weiss NM, Erhard V, et al. A novel three-step process for the identification of inner ear malformation types. *Laryngoscope Investig Otolaryngol*. Dec 2022;7(6):2020-2028. doi:10.1002/lio2.936

69. Di Maro F, Carner M, Sacchetto A, Soloperto D, Marchioni D. Frequency reallocation based on cochlear place frequencies in cochlear implants: a pilot study. *Eur Arch Otorhinolaryngol*. Oct 2022;279(10):4719-4725. doi:10.1007/s00405-021-07245-y

70. Dutrieux N, Quatre R, Pean V, Schmerber S. Correlation Between Cochlear Length, Insertion Angle, and Tonotopic Mismatch for MED-EL FLEX28 Electrode Arrays. *Otol Neurotol*. Jan 1 2022;43(1):48-55. doi:10.1097/MAO.0000000000003337

71. Kurz A, Muller-Graff FT, Hagen R, Rak K. One Click Is Not Enough: Anatomy-Based Fitting in Experienced Cochlear Implant Users. *Otol Neurotol*. Dec 1 2022;43(10):1176-1180. doi:10.1097/MAO.0000000000003731

72. Li J, Kang S, Du H, et al. Analysis of Cochlear Parameters in Paediatric Inner Ears with Enlarged Vestibular Aqueduct and Patent Cochlea. *J Pers Med*. Oct 7 2022;12(10)doi:10.3390/jpm12101666

73. Mertens G, Van de Heyning P, Vanderveken O, Topsakal V, Van Rompaey V. The smaller the frequency-to-place mismatch the better the hearing outcomes in cochlear implant recipients? *Eur Arch Otorhinolaryngol*. Apr 2022;279(4):1875-1883. doi:10.1007/s00405-021-06899-y

74. Muller-Graff FT, Ilgen L, Schendzielorz P, et al. Implementation of secondary reconstructions of flat-panel volume computed tomography (fpVCT) and otological planning software for anatomically based cochlear implantation. *Eur Arch Otorhinolaryngol*. May 2022;279(5):2309-2319. doi:10.1007/s00405-021-06924-0

75. Thimsen V, Mantsopoulos K, Liebscher T, et al. Association between lateral wall electrode array insertion parameters and audiological outcomes in bilateral cochlear implantation. *Eur Arch Otorhinolaryngol*. Jun 2023;280(6):2707-2714. doi:10.1007/s00405-022-07756-2

76. Topsakal V, Heuninck E, Matulic M, et al. First Study in Men Evaluating a Surgical Robotic Tool Providing Autonomous Inner Ear Access for Cochlear Implantation. *Front Neurol*. 2022;13:804507. doi:10.3389/fneur.2022.804507

77. Al-Dhamari I, Helal R, Abdelaziz T, Waldeck S, Paulus D. Automatic cochlear multimodal 3D image segmentation and analysis using atlas-model-based method. *Cochlear Implants Int*. Jan 2024;25(1):46-58. doi:10.1080/14670100.2023.2274199

78. Alahmadi A, Abdelsamad Y, Almuhawas F, Hamed N, Salamah M, Alsanosi A. Cochlear Implantation: The Volumetric Measurement of Vestibular Aqueduct and Gusher Prediction. *J Pers Med*. Jan 19 2023;13(2)doi:10.3390/jpm13020171

79. Alshalan A, Almuhawas F, Alhabib S, Hamed N, Abdelsamad Y, Dhanasingh A. Method to estimate the basal turn length in inner ear malformation types. *Sci Rep*. Jan 5 2023;13(1):66. doi:10.1038/s41598-022-23911-5

80. Breitsprecher T, Mlynski R, Volter C, et al. Accuracy of Preoperative Cochlear Duct Length Estimation and Angular Insertion Depth Prediction. *Otol Neurotol*. Sep 1 2023;44(8):e566-e571. doi:10.1097/MAO.0000000000003956

81. Geiger S, Iso-Mustajarvi M, Nauwelaers T, et al. Automatic electrode scalar location assessment after cochlear implantation using a novel imaging software. *Sci Rep*. Jul 31 2023;13(1):12416. doi:10.1038/s41598-023-39275-3

82. Hajr E, Abdelsamad Y, Almuhawas F, Alashour A, Hagr A. Cochlear Implantation: The use of OTOPLAN Reconstructed Images in Trajectory Identification. *Ear Nose Throat J*. Jan 7 2023:1455613221134742. doi:10.1177/01455613221134742

83. Hussain R, Frater A, Calixto R, et al. Anatomical Variations of the Human Cochlea Using an Image Analysis Tool. *J Clin Med*. Jan 8 2023;12(2)doi:10.3390/jcm12020509

84. Matin-Mann F, Gao Z, Wei C, et al. Development and In-Silico and Ex-Vivo Validation of a Software for a Semi-Automated Segmentation of the Round Window Niche to Design a Patient Specific Implant to Treat Inner Ear Disorders. *J Imaging*. Feb 20 2023;9(2)doi:10.3390/jimaging9020051

85. Mewes A, Bennett C, Dambon J, Brademann G, Hey M. Evaluation of CI electrode position from imaging: comparison of an automated technique with the established manual method. *BMC Med Imaging*. Sep 29 2023;23(1):143. doi:10.1186/s12880-023-01102-6

86. Paouris D, Kunzo S, Goljerova I. Validation of Automatic Cochlear Measurements Using OTOPLAN((R)) Software. *J Pers Med*. May 8 2023;13(5)doi:10.3390/jpm13050805

87. Rader T, Schrank L, Spiegel JL, et al. Comparison of speech perception in bimodal cochlear implant patients with respect to the cochlear coverage. *HNO*. Jan 2024;72(Suppl 1):17-24. doi:10.1007/s00106-023-01327-5

88. Sieber D, Timm ME, Weller T, Suhling M, Lenarz T, Schurzig D. The Dependency of Cochlear Lateral Wall Measurements on Observer and Imaging Type. *Otol Neurotol*. Oct 1 2023;44(9):873-880. doi:10.1097/MAO.0000000000003991

89. Swarup A, Karakkandy V, Chappity P, et al. Comparing accuracy of cochlear measurements on magnetic resonance imaging and computed tomography: A step towards radiation-free cochlear implantation. *J Otol*. Oct 2023;18(4):208-213. doi:10.1016/j.joto.2023.08.001

90. Tavora-Vieira D, Voola M, Kuthubutheen J, Friedland P, Gibson D, Acharya A. Evaluation of the Performance of OTOPLAN-Based Cochlear Implant Electrode Array Selection: A Retrospective Study. *J Pers Med*. Aug 20 2023;13(8)doi:10.3390/jpm13081276

91. Alshalan A, Abdelsamad Y, Alahmadi A, et al. Estimation of outer-wall length in optimizing cochlear implantation in malformed inner ears. *Sci Rep*. Nov 9 2024;14(1):27308. doi:10.1038/s41598-024-77991-6

92. Dessard L, Gersdorff G, Ivanovik N, et al. Cochlear Implant: Analysis of the Frequency-to-Place Mismatch with the Table-Based Software OTOPLAN(R) and Its Influence on Hearing Performance. *Audiol Neurootol*. 2024;29(3):239-245. doi:10.1159/000535693

93. Dhanasingh A, Nielsen SB, Beal F, et al. Cochlear implant electrode design for safe and effective treatment. *Front Neurol*. 2024;15:1348439. doi:10.3389/fneur.2024.1348439

94. Jun B, Song S. Analysis of Cochlear Morphology for Cochlear Implantation Using Three-Dimensional Reconstruction of Computed Tomography Images. *Audiol Neurootol*. 2024;29(3):207-215. doi:10.1159/000534739

95. Spahn B, Ilgen L, Neun T, et al. Dependence of Cochlear Duct Length Measurement on the Resolution of the Imaging Dataset. *Otol Neurotol*. Mar 1 2024;45(3):e234-e240. doi:10.1097/MAO.0000000000004088
